# Fibroblast growth factor receptor 1 (FGFR1) as a therapeutic target in adenoid cystic carcinoma of the lacrimal gland

**DOI:** 10.18632/oncotarget.26558

**Published:** 2019-01-11

**Authors:** Ravi Doddapaneni, Wensi Tao, Andrea Naranjo, Neda Nikpoor, David T. Tse, Daniel Pelaez

**Affiliations:** ^1^ Dr. Nasser Al-Rashid Orbital Vision Research Center, Bascom Palmer Eye Institute, Department of Ophthalmology, University of Miami Miller School of Medicine, Miami, FL 33136, USA; ^2^ Department of Biomedical Engineering, University of Miami Miller School of Medicine, Miami, FL 33136, USA; ^3^ Department of Cell Biology, University of Miami Miller School of Medicine, Miami, FL 33136, USA; ^4^ Sylvester Comprehensive Cancer Center, University of Miami Miller School of Medicine, Miami, FL 33136, USA

**Keywords:** adenoid cystic carcinoma, lacrimal gland, FGFR, targeted therapy, precision medicine

## Abstract

Identification of molecular targets is the first step in developing efficacious therapeutic strategies for tumors. A tumors' biological response to perturbagens yields important information on the molecular determinants for tumor growth. The aim of this study was to characterize the response of adenoid cystic carcinoma of the lacrimal gland (LGACC) to intra-arterial cytoreductive chemotherapy (IACC) in order to identify novel targets to enhance therapy. We performed high-throughput proteomic analysis on paired samples from pre-IACC diagnostic biopsies and post-IACC excised tumor samples from 6 LGACC patients. This proteomic analysis provides a comprehensive landscape of the cellular compartments contained within the excised tumors. Interestingly, we found a strong upregulation across the fibroblast growth factor (FGF) signaling pathway, with FGF receptor 1 (FGFR1) exhibiting a consistent and significant upregulation in all post-IACC samples. We thus evaluated the therapeutic efficacy of a novel FGFR1 selective inhibitor, AZD4547, in combination with cisplatin on LGACC cells in-vitro. The combination index (CI) value (<0.895) demonstrated synergistic effect of AZD4547 and cisplatin in inhibiting cell growth and viability (p<0.02), with a differential response seen in post-IACC cultures when compared to pre-IACC cultures. The combination approach showed synergy of the drugs in the migration assay. Western blot analysis indicated a significant upregulation of cleaved caspase-3 and downregulation the expression of FGFR1 (p<0.05) with the combination treatment as compared to either agent independently. Our findings demonstrate that FGFR1 inhibition potentiates the cytoreductive effects of cisplatin and suggest a potential therapeutic benefit of using AZD4547 in the management of LGACC.

## INTRODUCTION

Precision medicine and targeted therapies exploit the biological nuances that distinguish pathological from healthy tissues in order to achieve effective medical management of tumors while limiting off-target effects. Adenoid cystic carcinoma (ACC) is the most common malignant epithelial cancer of the lacrimal gland. ACC of the lacrimal gland is a challenging disease to manage due to a high recurrence rate, propensity for intracranial invasion, and poor prognosis [1–3]. The slow growth pattern of LGACC hinders the assessment of systemic therapy efficacy in metastatic disease and the 15-year survival rate has been reported to be as low as 20% [4]. Currently, the standard of care for locally advanced lacrimal gland ACC is orbital exenteration followed by postoperative chemo-radiation therapy [5–7]. However, there are no reliable or targeted therapeutic options for long-term disease control of LGACC [7–9]. The most novel treatment proposed to date is the incorporation of intra-arterial cytoreductive chemotherapy (IACC) in lacrimal gland ACC [10, 11]. Although results with this therapy have been encouraging in downstaging the disease to surgical resectability, non-specific drug toxicities and poorly understood resistance mechanisms have been observed in patients who develop local and regional recurrences, or who eventually succumb to metastatic disease. Due to the low incidence of LGACC, the molecular mechanisms underlying tumor progression, metastasis, and resistance to treatment are underexplored. This is a major roadblock for understanding the biology and development of an effective targeted therapy for treatment of LGACC. In general, in the absence of targeted tumor management therapies, accepted treatment strategies are thought to fail because of multidrug resistance (MDR) and adaptability phenomena [12]. Therefore, combination therapies of chemotherapeutic drugs with a targeted approach have been considered to overcome drug-resistance, reduce off-target toxicity and improve clinical outcomes.

Recently, molecular and exome-sequencing studies have begun to elucidate the molecular landscape of LGACC in an effort to understand its pathophysiology and potentially alter its clinical course [13]. These studies have identified the mutational profile of LGACC which are analogous to other ACC lesions of the head and neck, including the existence of high-penetrance alterations of the proto-oncogene MYB, as well as mutually-exclusive mutations in the Wnt, PTEN, or Notch pathways. Activating Notch mutations have been found to correlate to the most aggressive phenotype in LGACC, basaloid undifferentiated solid pattern tumors, and are a major focus of ongoing research. However, there are currently no viable therapeutic agents to effectively target either MYB or Notch activated signaling. Similarly, it has been shown that targeted agents against epidermal growth factor receptor (EGFR) (cetuximab, erlotinib, and gefitinib), other receptor tyrosine kinases (RTK) (imatinib and dovitinib), and histone deacetylases (HDAC) (vorinostat) have limited activity against ACCs [14–18], highlighting the need for a disease-context specific approach to the identification of therapeutic targets for given tumors.

To that end, the use of cellular perturbation response datasets has recently gained impetus in the oncology field and has led to the establishment of bioinformatic tools such as the Library of Integrated Network-based Cellular Signatures (LINCS), led by the NIH [19]. Analyzing the cellular and tissue response to a given perturbagen can yield information about the cellular processes and pathways engaged to subvert the effects of the modulating agent and the critical elements required for this response. In no other context is perturbation response analysis more clinically-actionable than in the analysis of an actual tumor tissue response to standard medical management. This analysis gains more strength if the differential comparison can be performed in a paired sample fashion, where confounding parameters such as background genetic variability of different patients can be eliminated. Thus, the aim of this study was to perform a proteomic high throughput response analysis of available paired LGACC samples before and after treatment with intra-arterial cytoreductive chemotherapy (IACC cisplatin and doxorubicin), with the goal of identifying novel therapeutic markers that could, enhance our clinical long-term control of the LGACC patient.

## RESULTS

### Differential protein profile following chemotherapeutic challenge in LGACC

**Figure 1:**
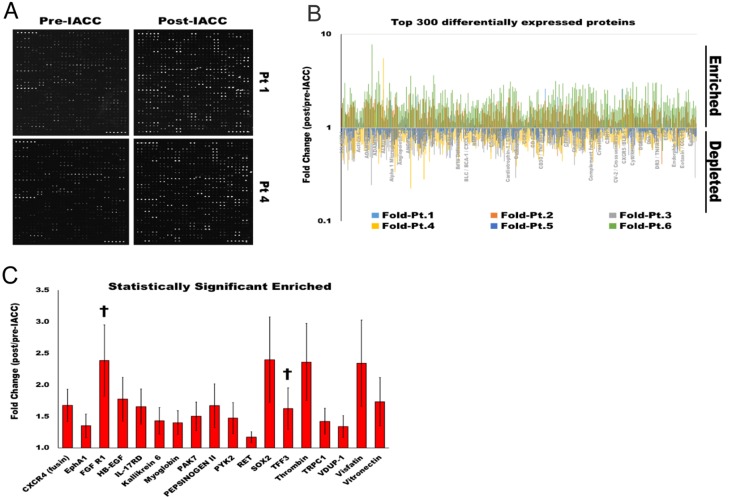
Proteomic profile of LGACC tumor's response to IACC protocol **(A)** Representative image of L-series array immunoblot readout for pre-, and post-IACC paired samples from 2 individual LGACC patients. **(B)** Log fold representation of paired proteomic readout for the top 300 differentially expressed protein for all 6 individual patients showing enriched (>1.0), and depleted (<1.0) proteins in post-IACC samples. **(C)** Bar graph of statistically significant enriched proteins in aggregated post/pre-IACC data for all 6 patients († proteins with statistically significant enrichment in all 6 individual paired patient specimens). (p<0.05).

Out of the 1,000 proteins in the proteomic array used in this study, we obtained quantifiable readouts for 829 proteins after quality control filtering in our samples (Figure 1A). Within this readout, 133 proteins were depleted or downregulated in post-IACC samples, while a total of 696 proteins were found to be enriched or upregulated in the post-IACC samples when compared to their paired pre-IACC specimens (Figure 1B). Functional enrichment analysis of biological processes represented by the top 300 enriched proteins in post-IACC samples shows intracellular signaling cascades, membrane-bound receptor signaling, and development as the top 5 significantly represented biological processes when filtered by false discovery rate (FDR) (Table 1). We identified a total of 18 proteins that were enriched in a statistically significant manner in the pooled data for all 6 post-IACC patient samples (Figure 1C). Some of the proteins that were enriched in post-IACC samples in our screen have previously been described in LGACC as potential therapeutic markers, such as c-kit (SCFR/CD117) [13]. However, most of these markers showed high variability and inconsistencies between the pairs of samples in our cohort. Specifically, for c-kit expression, only 2/6 (33.33%) samples showed a significant upregulation of this marker following IACC, while 3/6 (50%) showed no difference in c-kit expression between pre- and post-IACC, and 1/6 (16.67%) actually had a downregulation of this specific marker. This suggests that this marker may not be a significant target to enhance treatment response in general, and may be a more appropriate readout for a personalized medicine approach.

**Table 1:** Ontology for biological processes represented in the top 300 enriched proteins identified in post-IACC samples

Biological Process	REFLIST	Protein Hits	Expected	Fold Enrichment	Raw P-value	FDR
**MAPK cascade (GO:0000165)**	340	34	5.36	6.34	1.15E-16	2.81E-14
**intracellular signal transduction (GO:0035556)**	1071	53	16.9	3.14	4.26E-13	5.20E-11
**signal transduction (GO:0007165)**	2318	83	36.57	2.27	1.91E-12	1.55E-10
**cell surface receptor signaling pathway (GO:0007166)**	1206	55	19.03	2.89	3.22E-12	1.97E-10
**developmental process (GO:0032502)**	1501	62	23.68	2.62	6.31E-12	3.08E-10
**response to stimulus (GO:0050896)**	2677	87	42.24	2.06	5.37E-11	2.19E-09
**negative regulation of apoptotic process (GO:0043066)**	99	15	1.56	9.6	2.81E-10	9.79E-09
**regulation of catalytic activity (GO:0050790)**	359	26	5.66	4.59	4.05E-10	1.24E-08
**cell communication (GO:0007154)**	2686	85	42.38	2.01	5.07E-10	1.38E-08
**transmembrane receptor PTK signaling pathway (GO:0007169)**	151	17	2.38	7.14	1.17E-09	2.86E-08
**regulation of phosphate metabolic process (GO:0019220)**	537	30	8.47	3.54	5.81E-09	1.18E-07
**regulation of molecular function (GO:0065009)**	441	27	6.96	3.88	5.69E-09	1.26E-07
**immune system process (GO:0002376)**	669	33	10.56	3.13	1.76E-08	3.31E-07
**apoptotic process (GO:0006915)**	336	22	5.3	4.15	5.09E-08	8.87E-07
**immune response (GO:0006955)**	383	23	6.04	3.81	1.10E-07	1.79E-06
**cell death (GO:0008219)**	356	22	5.62	3.92	1.32E-07	1.89E-06
**death (GO:0016265)**	356	22	5.62	3.92	1.32E-07	2.01E-06
**cell differentiation (GO:0030154)**	548	25	8.65	2.89	3.86E-06	4.70E-05
**biological regulation (GO:0065007)**	2985	79	47.1	1.68	3.82E-06	4.90E-05
**locomotion (GO:0040011)**	310	18	4.89	3.68	4.24E-06	4.92E-05
**response to external stimulus (GO:0009605)**	351	19	5.54	3.43	5.99E-06	6.64E-05
**regulation of biological process (GO:0050789)**	2463	67	38.86	1.72	1.05E-05	1.07E-04
**cytokine-mediated signaling pathway (GO:0019221)**	60	8	0.95	8.45	1.02E-05	1.09E-04
**biological adhesion (GO:0022610)**	356	16	5.62	2.85	2.55E-04	2.31E-03
**cell adhesion (GO:0007155)**	356	16	5.62	2.85	2.55E-04	2.40E-03
**angiogenesis (GO:0001525)**	18	4	0.28	14.08	3.35E-04	2.82E-03
**cellular component movement (GO:0006928)**	480	19	7.57	2.51	3.27E-04	2.85E-03
**protein phosphorylation (GO:0006468)**	81	7	1.28	5.48	4.43E-04	3.60E-03
**cell-cell adhesion (GO:0016337)**	141	9	2.22	4.05	5.77E-04	4.40E-03
**protein folding (GO:0006457)**	94	7	1.48	4.72	1.01E-03	7.48E-03
**response to stress (GO:0006950)**	653	22	10.3	2.14	1.19E-03	8.32E-03
**proteolysis (GO:0006508)**	448	17	7.07	2.41	1.68E-03	1.11E-02
**cell-matrix adhesion (GO:0007160)**	51	5	0.8	6.21	1.75E-03	1.13E-02
**cellular calcium ion homeostasis (GO:0006874)**	115	7	1.81	3.86	2.99E-03	1.82E-02
**response to interferon-gamma (GO:0034341)**	58	5	0.92	5.46	2.95E-03	1.85E-02
**cellular process (GO:0009987)**	8247	157	130.12	1.21	3.19E-03	1.90E-02
**behavior (GO:0007610)**	61	5	0.96	5.2	3.61E-03	2.10E-02
**induction of apoptosis (GO:0006917)**	41	4	0.65	6.18	5.16E-03	2.86E-02
**ectoderm development (GO:0007398)**	212	9	3.34	2.69	7.90E-03	4.10E-02

To analyse our proteomic data, we used Gene PANTHER (Protein ANnotation THrough Evolutionary Relationships) classification system (http://www.pantherdb.org/) that combines gene function, ontology, pathways and statistical analysis tools to analyze large-scale, genome-wide data from proteomics experiments. The False Discovery Rate (FDR) adjusts the value at which a test is considered “significant” based on the rank of the predicted level of significance.

**Figure 2:**
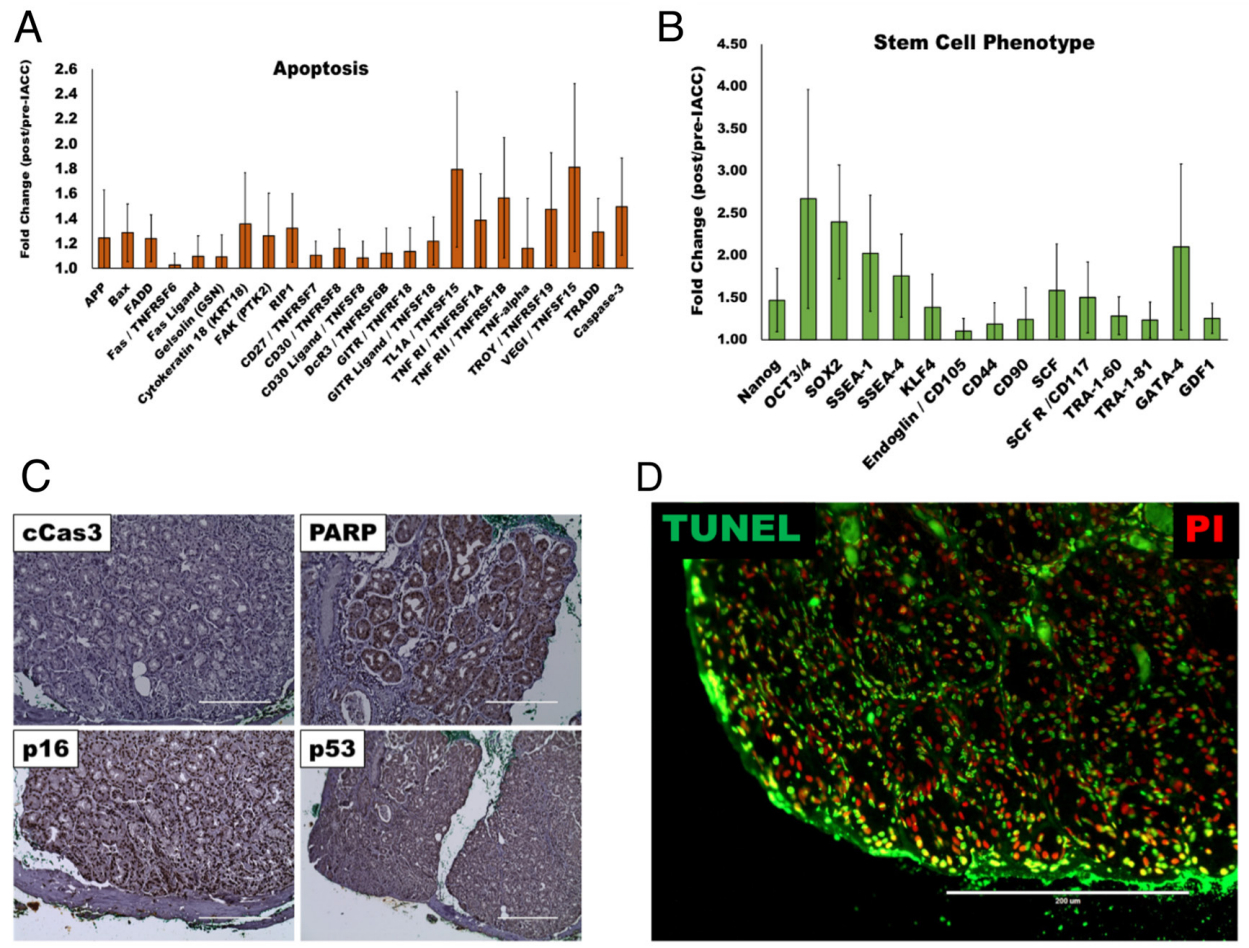
Apoptotic and stem/progenitor phenotypes present in post-IACC tumor samples **(A)** Bar graph for apoptotic cell marker cluster identified as enriched in aggregate post/pre-IACC data in our proteomic data (statistically significant, p<0.05) **(B)** Bar graph of lacrimal gland stem/progenitor cell markers identified as enriched in aggregate post/pre-IACC samples in our proteomic data (statistically significant, p<0.05). **(C)** Immunohistochemical staining representative for apoptotic markers cleaved caspase 3 (cCas3), Poly (ADP-ribose) polymerase-1 (PARP-1), p16, and p53 in post-IACC LGACC tumors. **(D)** Immunofluorescence image for terminal deoxynucleotidyl transferase (TdT) dUTP nick-end labeling (TUNEL) assay in post-IACC LGACC specimens. (micron bar = 200 μm).

As expected, due to the nature of the chemotherapeutic treatment, most apoptotic markers assayed were found to be upregulated in the post-IACC tumor samples (Figure 2A). Similarly, biological process ontology identified ‘response to stimulus’ and ‘negative regulation of apoptotic process’ as the sixth and seventh most significantly represented cluster within the top 300 top enriched proteins in post-IACC samples (Table 1). Finally, histopathological staining for cleaved caspase 3, PARP, tumor suppressors p16, and p53, as well as an in-vitro assay for terminal deoxynucleotidyl transferase dUTP nick end labeling (TUNEL) confirmed a high degree of apoptotic cells present in post-IACC samples (Figure 2C, 2D). Interestingly, and in accordance with our bioinformatic clustering identifying ‘developmental process’ as an enriched cluster in post-IACC samples, we identified several proteins associated to a stem/progenitor cell phenotype that are upregulated following IACC (Figure 2B). Whether this data represents a cancer stem cell phenotype capable of recapitulating the primary lesion with all its heterogeneity is currently under investigation. Several of the identified proteins in this cluster were previously reported by our laboratory in the developing lacrimal gland [19] and strengthen the concept of a cancer stem cell population reactivating normal embryonic programs upon insults to the lacrimal gland.

### Fibroblast growth factor (FGF) signaling is upregulated in post-IACC LGACC

**Figure 3:**
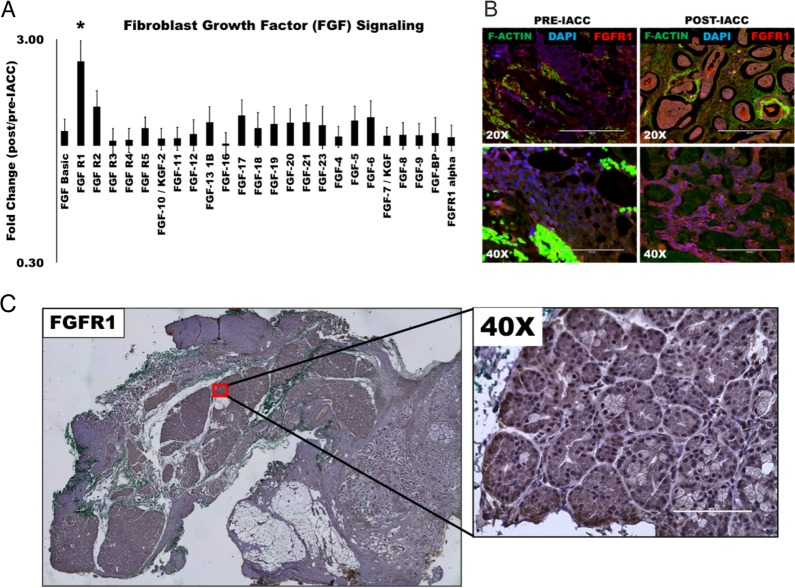
Fibroblast growth factor (FGF) signaling is upregulated following IACC in LGACC tumors **(A)** Bar graph of normalized quantitative fold expression of fibroblast growth factor (FGF) signaling family members in aggregate post/pre-IACC LGACC tumors identified in proteomic screening (statistically significant, p<0.05). **(B)** Immunofluorescence images of paired pre- and post-IACC LGACC samples probed for FGFR1 (red), and counterstained for filamentous actin (green), and nuclei (blue). **(C)** Representative immunohistochemical staining image for FGFR1 in a post-IACC LGACC tumor specimen. (micron bars: 20X=200 μm; 40X=100 μm).

A thorough analysis of our data revealed a strong signature of cell surface receptor signaling amongst enriched proteins in post-IACC samples, in particular receptor tyrosine kinases (RTK). In fact, all the members in the Fibroblast Growth Factor (FGF) signaling pathway assayed were elevated in the post-IACC samples (Figure 3A) with FGF-Receptor 1 (FGFR1) being one of only two proteins in the entire array that was statistically significantly enriched consistently in all 6 paired patient samples (Figure 1C, †) (p = 0.029). This observation was further validated by immunofluorescence and immunohistochemistry staining in all samples (Figure 3B, 3C). Conversely, FGF receptor 2 (FGFR2) was significantly upregulated or enriched in 4 out of 6 of our patient samples yet did not reach statistical significance in the combined patient data (p=0.064). Similarly, FGF17 was significantly enriched in 5 out of 6 post-IACC paired patient samples, but was significantly depleted in the 1 out of 6 samples, leaving the overall significance below the p=0.05 cutoff (p=0.093). Our immunofluorescence and immunohistochemistry analysis demonstrates a high degree of FGFR1 expression in the solid epithelial cell clusters of post-IACC tumor tissues (Figure 3B, 3C), which reinforced our proteomic data finding and led us to further investigate this marker in LGACC. We thus evaluated a novel and selective FGFR1 tyrosine kinase inhibitor, AZD4547, which is under phase II/III clinical trials in combination with standard chemotherapeutic drug cisplatin, for targeting LGACC *in vitro*.

### LGACC cell lines retain primary tumor phenotypes and show a differential response to FGFR1 inhibition

**Figure 4:**
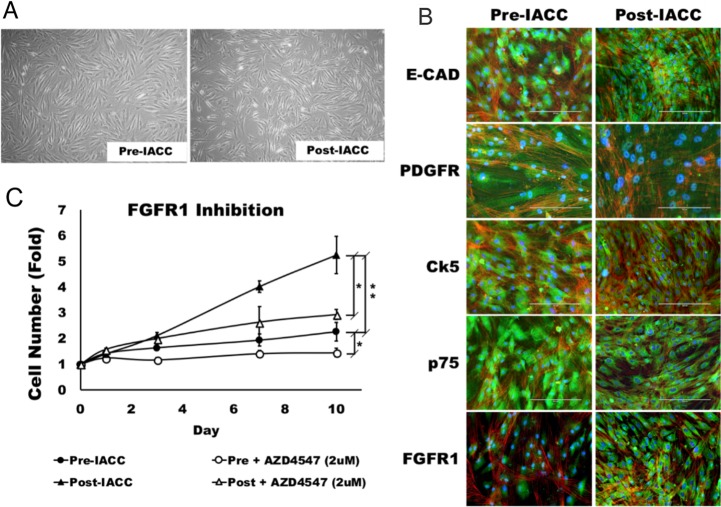
Morphology and characterization of LGACC cell cultures **(A)** Morphology of established LGACC cell lines from primary tumors from pre- and –post IACC samples cultured in serum free human mammary epithelial cell media **(B)** Characterization of LGACC cells by immunocytochemistry using probes for markers e-cadherin (E-CAD), platelet-derived growth factor receptor (PDGFR), cytokeratin-5 (Ck5), low affinity neurotrophin receptor (p75), and fibroblast growth factor receptor 1 (FGFR1). **(C)** Growth curve over a 10 day period for pre- and post-IACC cell cultures in control or FGFR1 inhibitor-supplemented media using AZD4547 (2μM). (micron bar = 200 μm).

Cell lines were established from pre- and post-IACC samples of LGACC (Figure 4A). These cells were characterized immunocytochemically for standard LGACC markers E-cadherin (E-cad), cytokeratin-5 (Ck-5), platelet –derived growth factor receptor (PDGFR) and the low affinity neurotrophin receptor (p75) (Figure 4B). Finally, cells were probed for their expression of FGFR1 which showed an increased level of expression in post-IACC cell cultures when compared to pre-IACC lines (Figure 4B). Cells were characterized for their growth characterisitics, which demonstrated that, much like the progression of LGACC tumors, these cells have low proliferative activity with doubling times greater than 120 hrs. for pre-IACC cells. Cells from post-IACC samples showed a significantly faster proliferative rate than pre-IACC cells with doubling times of 48hrs (Figure 4C, p<0.01). Supplementation of culture media with the FGFR1 inhibitor AZD4547 (2μM) resulted in a statistically significant decrease in cellular proliferative rates for both pre-, and post-IACC cell lines, with post-IACC cells exhibiting a greater differential response to this inhibition than pre-IACC cells (Figure 4C).

### FGFR1 inhibition and cisplatin synergize to enhance cellular cytotoxic effects

**Figure 5:**
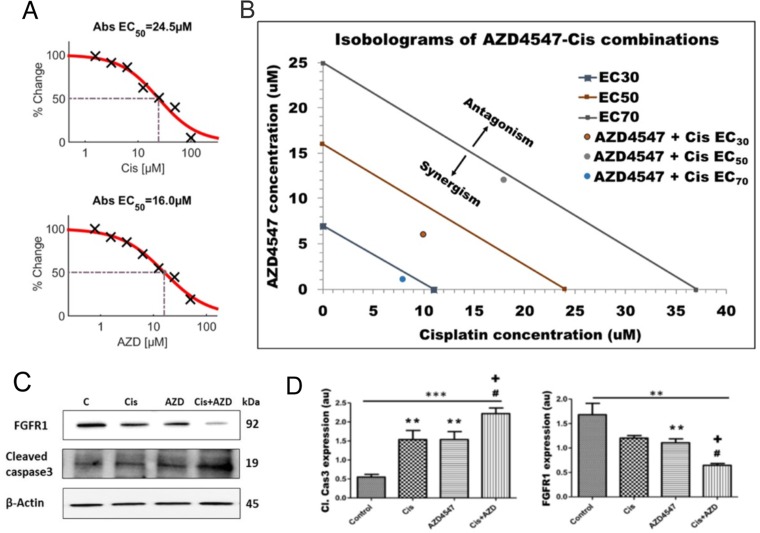
Synergistic effect of AZD4547 and cisplatin on LGACC cell viability **(A)** Dose-dependent viability curves showing the half-maximal effective dose (EC_50_) for cisplatin and AZD4547. **(B)** Isobologram of synergistic cooperation between cisplatin and AZD4547 on LGACC cell viability (points plotted) over a 72 hrs. period. **(C)** Western blot analysis of LGACC cells following treatment with cisplatin, AZD4547, and their combination. **(D)** The quantitative expression of indicated proteins as determined by densitometric analysis. Data are calculated from triplicate experiments and presented as mean±SD (^**^p<0.05; ^***^p<0.01 compared with control; ^#^p<0.05 wrt to cisplatin; ^+^p<0.05 wrt to AZD4547).

In order to investigate whether FGF signaling represents an adaptive or resistant pathway for LGACC in response to IACC with cisplatin, we performed cellular viability assays as a function of drug concentrations for both cisplatin and AZD4547 independently to determine the half maximal effective dose (EC_50_) on LGACC cell viability (Figure 5A). Our results show that both cisplatin and AZD4547 inhibit the viability of LGACC cells over a 72hr period in a dose-dependent manner, with an EC_50_ of 24.5μM and 16.0μM respectively. We further analyzed the synergism between cisplatin and AZD4547 via isobologram and combination index measurements (Figure 5B). The combination of AZD4547 and cisplatin showed significantly higher inhibition rate when compared with single-agent treatments across the multiple LGACC cell lines. The combination index was calculated to be 0.895 and is represented by the data points plotted, which all fall below the additivity lines (straight line connecting the EC_30_, EC_50_ and EC_70_ values of AZD4547 and cisplatin), indicating a synergistic effect on cell viability. Classical isobolograms were shown in Figure 5B, the results indicates that points of combination of AZD4547 and cisplatin is located below the line (synergy) at EC_30_, EC_50_ and EC_70_. We further used western blot analysis to assess the expression of the apoptotic marker cleaved caspase 3, and FGFR1 following single drug, and combination treatments. Our data shows that both cisplatin and AZD457 significantly increase the levels of cleaved caspase 3 when compared to control, but that the combinatorial treatment further increases the apoptotic marker significantly over either drug alone (Figure 5C, 5D). Conversely, only AZD4547 shows a significant decrease in the expression of FGFR1 when compared to control, but that the combination treatment significantly enhances this effect over the single agent supplementation (Figure 5C, 5D).

### The combinatorial approach of cisplatin treatment and FGFR1 inhibition restricts LGACC cell migration and proliferation

**Figure 6:**
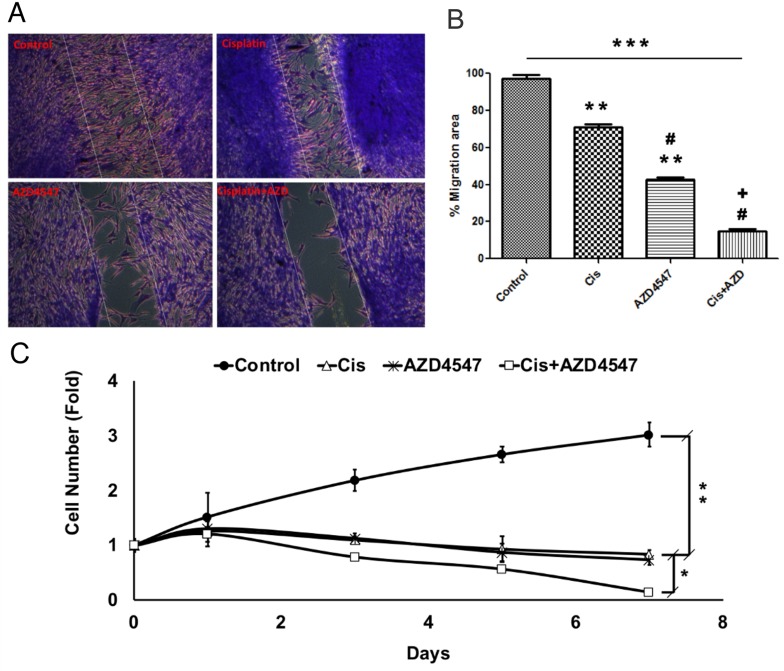
Synergistic effect of AZD4547 and cisplatin on LGACC cell migration and proliferation **(A)** Representative images of migration assay results for LGACC cells after 48 hrs. of control, cisplatin, AZD4547, or combinatorial treatment. **(B)** Quantitative analysis of gap bridging percentage in migration assay of LGACC cells under different treatments. **(C)** Growth curve analysis of LGACC cells over 7 days of control, cisplatin, AZD4547, or combinatorial treatment. Data presented as mean±SD (^*^p<0.05; ^**^p<0.01; ^***^p<0.001 compared with control; ^#^p<0.01 wrt to cisplatin; ^+^p<0.01 wrt to AZD4547).

In order to assess the effect of cisplatin, AZD4547, and the combinatorial treatment on the migratory and proliferative potential of LGACC cells, we performed scratch and MTT assays, on available cell lines. Results of the scratch wound-healing assay (Figure 6A) showed that LGACC cells under control conditions exhibited a >95% (97.5 ± 1.25%) bridging of the acellular gap over a 48hr period, whereas LGACC cells treated with cisplatin alone (12.25 μM) had a 68.7 ± 3.12% closure (Figure 6B, ^**^p<0.01 wrt controls), and cells treated with AZD4547 (8 μM) alone exhibited a 38.5 ± 1.57% (Figure 6B, ^**^p<0.01wrt controls) bridging. Interestingly, the migratory activity of cells treated with AZD4547 alone was significantly decreased when compared to those treated with cisplatin alone (Figure 6B, ^#^p<0.01). However, cells treated with the combination of cisplatin and AZD4547 had a gap closure of only 24.5 ± 2.05% which represented a statistically significant decrease in migratory activity when compared to controls (p<0.001), as well as to the cisplatin (^#^p<0.01) and AZD4547 (^+^p<0.01) single-agent treatment groups (Figure 6B). Similarly, the results of our MTT cell proliferation assay demonstrated that either cisplatin (12.25 μM), or AZD4547 (8 μM) supplementation alone were enough to effectively inhibit cell growth in the LGACC cell lines when compared to control cell growth over a 7-day period (Figure 6C, ^**^p<0.01). Yet, in terms of cell proliferation, the combination approach of cisplatin and AZD4547 treatment was shown to not only inhibit cell growth, but actually show a decrease in overall cell numbers over the 7-day period, corroborating our finding of the synergistic effect on cellular viability in the combinatorial treatment. This effect was significant when compared to cell proliferation with either drug alone (Figure 6C, p<0.05). Taken together, these results suggest that combination therapy may result in various synergistic anti-tumor effects on LGACC tumors.

## DISCUSSION

The development of targeted and effective cancer therapy is often plagued by discrepancies between drug efficacies obtained in preclinical studies and the outcomes of clinical trials. These inconsistencies can be largely attributed to a lack of clinical relevance of the in-vitro assays and models used for therapeutic target identification. In this study, we focused on characterizing the response from paired lacrimal gland adenoid cystic carcinoma (LGACC) tumor specimens to the most advanced modality of medical management in order to elucidate the mechanisms employed by the tumor to subvert the effect of treatment. Adenoid cystic carcinoma is the most common epithelial malignancy of the lacrimal gland and, although rare, has a poor prognosis when managed with conventional treatment [6, 20]. In 1998, Meldrum et al [10] reported the first case series of two patients in which the novel approach of local high-dose cisplatin delivered via intracarotid cannulation of the lacrimal artery was evaluated. Given the results obtained in this study demonstrating tumor down-staging to surgically resectable lesions, and the rate of local control of observed, a subsequent study by Tse et al [21] evaluated this novel approach, now known as intra-arterial cytoreductive chemotherapy (IACC), on an additional nine patients and compared it to historical controls receiving conventional local therapies. This study demonstrated that IACC resulted in improved local disease control and overall disease-free survival, effectively changing the management paradigm for the disease. Yet despite these encouraging advancements in local management, patients still undergo orbital exenteration as part of their treatment, and many eventually experience recurrences and succumb to metastatic disease [22, 23]. We thus sought to investigate how these tumors respond to the IACC protocol and elucidate the cellular phenotypes that are capable of surviving the treatment regimen by taking advantage of a unique resource available at our institution, the largest repository of pre-IACC diagnostic biopsies, and post-IACC exenteration specimens from the same patient. This paired sampling modality allows for the nuanced evaluation of the tumors' response to IACC treatment without confounding environmental, lifestyle, or genetic variabilities.

Our results provide the first proteomic profile of the differential phenotype elicited by high-dose local chemotherapeutic challenge to LGACC tumors. As was expected, one of the strongest signatures found in the data was one of an apoptotic response in post-IACC samples, which was corroborated by histopathological examination of the tissue sections and had also been reported by Tse et al [21]. Yet one of the most interesting pieces of data gleaned from our profiling comes from consistent and robust responses in particular protein markers in post-IACC samples. Our data contains 2 such proteins in which each sample pair showed a significant increase in the post-IACC sample, the Trefoil Factor 3 (TFF3), and the Fibroblast Growth Factor Receptor 1 (FGFR1). While little is known about TFF3 in the context of adenoid cystic carcinoma, there are reports indicating that it is involved in epithelial tissue healing and restitution [24], which may explain the role for its high expression following injurious stimuli to LGACC tumors. Yet, the lack of specific inhibitors and the extracellular manifestation of this protein make it not amenable for therapeutic targeting.

On the other hand, FGF signaling has been widely studied in many organ systems and tumors [25, 26]. In our dataset, all FGF family members investigated were enriched in post-IACC samples, with FGFR1 being significantly upregulated in all 6 post-IACC specimens consistently. FGF signaling is known to play an important role during lacrimal gland embryonic development [19, 27, 28], and according to our data, may represent the main pathway for survival, growth, and chemoresistance utilized by LGACC tumors. In fact, our data also shows a strong signature corresponding to a stem/progenitor cell phenotype in post-IACC samples, further strengthening the idea that LGACC recapitulate embryonic developmental programs in order to overcome the cytotoxic effects of chemotherapy. Similarly, our in-vitro data demonstrates that FGFR1 is upregulated in LGACC cells even after several passages, indicating a more profound epigenetic reconfiguration being responsible for FGFR1 increased expression.

Importantly, our in-vitro assays confirm that FGFR1 inhibition with AZD4547 synergizes with cisplatin to sensitize LGACC cells and augment its cytoreductive effects. These assays were performed in patient-derived LGACC cell cultures, which constitutes the ideal in-vitro platform for such an investigation. And while in-vitro conditions introduce several artificial components to the environment for testing not seen in the in-vivo setting, the identification of the FGF pathway was achieved directly from human clinical samples, rather than in laboratory platforms. The in-vitro assays used only serve to corroborate and add strength to the clinical observation. Thus, this study constitutes a successful example of reverse translation of research from the bedside to the bench. Efforts are now being made to translate the in-vitro synergistic observations back to the bedside. We are working on establishing the preclinical animal models to perform these studies by xenograft of our established cell lines, patient-derived xenografts (PDX), or transgenic introduction of the LGACC driving mutations into mice LGs to promote endogenous LGACC formation. Similarly, there are ongoing clinical trials looking into the effect of receptor tyrosine kinase inhibitor Dovitinib, which has some affinity towards FGFR1-3 in the adenoid cystic carcinoma setting [29]. However, dovitinib is a multitargeted RTK inhibitor with its highest potency exerted on class III RTKs, FLT3 and c-Kit. On the other hand, AZD4547 was designed as a specific FGFR1/2/3 inhibitor with very low affinity for other RTKs, allowing for the distinction on the effect on the FGF pathway specifically without confounding off-target effects.

It is important to note that our findings only advance the use of FGFR1 inhibition strategies to enhance the cytoreductive effects of IACC, to cisplatin challenge in order to further enhance clinical control of LGACC progression. No evidence is presented to support that this is may represent a general response by epithelial tumors to chemotherapeutic challenges and further studies are needed to evaluate the extent to which this approach may be more broadly applied. Similarly, we must point out that our experimental design does not allow for the elucidation of whether this FGF-signaling signature represents an adaptavie mechanism by the tumor, or a resistive phenotype that is otherwise able to survive the chemotherapeutic regimen. We are currently performing tumor heterogeneity and clonality studies with single-cell RNA-seq to shed light on the mechanism for this enrichment.

## MATERIALS AND METHODS

### Ethics statement and LGACC tumor sample collection

Approval was obtained from the University of Miami Institutional Review Board (protocol #20090524) and all methods were compliant with the Declaration of Helsinki and the Health Insurance Portability and Accountability Act (HIPAA). Samples were collected from patients presenting with a lacrimal mass with a confirmed diagnosis of adenoid cystic carcinoma and who underwent the IACC protocol followed by orbital exenteration.

### Proteomic analysis

To examine protein profile of LGACC tissue samples, we compiled 6 patient samples for which we had the paired pre-IACC biopsy, and post-IACC resected specimens. We isolated full-length protein lysates using the Qproteome FFPE tissue specialized kit (Qiagen). Samples were then dialyzed to pure water in order to be run on the human L1000 membrane-based array (Ray Biotech), for unbiased semi-quantitative proteomic profiling. We analyzed data for upregulated or enriched proteins following chemotherapy.

### Immunohistochemistry

Immunohistochemistry was performed on LGACC tumor sections from our histopathology laboratory. Unstained sections were deparaffinized in xylene and re-hydrated on an ethanol gradient. Heat-mediated epitope retrieval was performed using sodium citrate buffer (pH 6.0) for 15 minutes. Samples were then blocked using 5% fetal bovine serum in PBS for 1 hour, then incubated overnight with primary antibodies against rabbit anti-cleaved caspase 3 (dilution 1:200, Cell signaling), rabbit anti-PARP (dilution 1:250, Bethyl Biosciences), mouse anti-p16 (dilution 1:200, Sigma), rabbit anti-p53 (dilution 1:500, GeneTex) and rabbit anti-FGFR1 (dilution 1:500, Cell signaling Inc). After washing in PBS 3 times, sections were incubated with species-specific secondary antibodies (immPress Reagent kit, Vector laboratories) for 2 hrs at room temperature, and developed using 3,3′-diaminobenzidine (DAB) colorimetric assay. Samples were counterstained with hematoxylin and eosin, mounted in aqueous mounting media and imaged.

### TUNEL assay

To confirm the degree of apoptotic cells present in LGACC tumors, LGACC post-IACC tumor sections were processed with the DeadEnd™ Fluorometric TUNEL System according to the manufacturer's instructions (Promega, Madison, WI). Briefly, the samples were deparrafinized, re-hydrated and then placed in assay equilibration buffer for 20 min, then incubated in a solution of terminal deoxynucleotidyl transferase (TdT) and fluorescein-12-dUTP-labeled DNA for 2 hrs. at 37°C. Reaction was stopped using a 2X saline sodium citrate (SSC) buffer. Nuclei were counterstained with propidium iodide solution, and samples were mounted and visualized on a Leica AOBS SP8 confocal microscope (Leica Microsystems, Exton, PA, USA).

### Establishment of LGACC cell lines

After proper consent, LGACC tissue was obtained from each patient. The tissue was aseptically dissected and minced using a scalpel and digested in a collagenase solution for 4 hr to obtain single cell suspension. The cells were then cultured on matrigel pre-coated flasks in presence of serum free human mammary epithelial cell media (HuMEC, Thermo Scientific, Waltham, MA) supplemented with hydrocortisone and pituitary bovine extract (Thermo Fisher Scientific), 100 U/mL penicillin and 100 mg/mL of streptomycin. When 70% confluency was reached, the cells were subcultured and serially passaged. Cell cultures were established for both pre-IACC (n=2), and post-IACC samples (n=4) when freshly available. Cells used in this study were all passage 9 or 10.

### Immunofluorescence

LGACC cells were seeded in 8-well chamber slides and cultured until confluent. Cells were then washed with PBS, and fixed for 10 minutes at room temperature with 4% paraformaldehyde (PFA). Cells were permeabilized for 5 minutes with 0.2% Triton X-100, and blocked for 30 minutes with 5% BSA. Cells were then incubated overnight at 4°C with primary antibodies against E-cadherin (250μg/ml, BD Biosciences), PDGFR (dilution 1:200, GeneTex), cytokeratin-5 (dilution 1:200, Abcam), FGFR1 (dilution 10μg/ml, abcam), and the low affinity neurotrophin receptor (p75, dilution 1:100, Abcam). After washing in PBS 3 times, sections were incubated with species-specific fluorescent secondary antibodies for 2 hr at room temperature. Finally, cells were counterstained for filamentous actin (F-actin, dilution 1:200) using AlexaFluor594 phalloidin (dilution 1:300, Thermofisher Scientific), and mounted with Vecta shield (Vector) fluorescent mounting medium containing DAPI. Imaging was performed with a Leica AOBS SP8 confocal microscope (Leica Microsystems Exton, PA, USA).

### Cell viability and synergistic studies

LGACC cells were maintained in culture flasks at 37°C in 5% CO_2_. The cells were seeded in 96-well plates (Eppendorf) at a density of 1×10^5^ cells per well in 100μL medium and were allowed to attach and grow for 24 hr. Afterwards, varying concentrations of cisplatin and AZD4547 were added to each well in order to generate dose response curves starting at a concentration of 1μM. The cells were incubated for 72 hr and cell viability was measured using the CellTiter-GLo assay (Promega, Madison, WI) following manufacturer's protocol. Luminescence measurements were performed on a SpectraMax i3 multimode plate reader (Molecular devices). Each concentration of cisplatin and AZD4547 was then plotted against percent cell survival. EC_50_ values were calculated compared to untreated control from 4-parameter fitted curves by solving for the X-intercept value at the 50% effect level of the Y-intercept value. Each treatment was performed in triplicate.

### Analysis of the combination index and drug interactions using the isobologram method

The combination index (CI) was calculated for the analysis of the synergistic, antagonistic or additive effects of the two drugs. The CI was calculated using the formula: C=[(D)_1_/(Dx)_1_] + [(D)_2_/(Dx)_2_], in which (D)_1_ was the concentration of the first drug required to achieve a particular effect in the combination; (Dx)_1_ was the concentration of the first drug that causes an identical effect alone; (D)_2_ was the concentration of the second drug that achieves a particular effect in the combination; (Dx)_2_ was the concentration of the second drug that generates the same effect alone. CI>1 indicates antagonism, CI=1 indicates an additive effect and CI<1 indicates synergy.

Classical isobolograms were also constructed by plotting drugs concentrations that effects 30%, 50%, 70% LGACC cell viability. First, the concentrations of AZD4547 and cisplatin required to produce a defined single-agent effect, when used as single agents, are placed on the x and y axes in a two-coordinate plot, corresponding to AZD4547 and cisplatin, respectively. The line connecting these two points was the line of additivity. Synergy, additivity, or antagonism are indicated when points are located below, on, or above the line, respectively.

### Immunoblotting

For immunoblot analysis, cells were treated with cisplatin, AZD4547 and the combination for 72 hr. Cells were washed twice with PBS and then lysed with RIPA cell lysis buffer (50 mM Tris-HCl, 150 mM NaCl, 0.1% SDS, 0.5% sodium deoxycholate, 1% NP40). Before SDS-PAGE, cell lysates were resuspended in SDS sample buffer (60 mmol/L Tris–HCl, 1% SDS, 10% glycerol, 0.05% bromophenol blue, pH 6.8, with 2% b-mercaptoethanol). Protein lysate was loaded onto a 4–20% gradient polyacrylamide gel (Bio-Rad) and then transferred to a PVDF membrane (Bio-Rad). PVDF membranes were incubated with blocking solution (TBS containing 0.1% Tween 20 and 3% BSA) and were probed with specific primary antibodies rabbit anti-FGFR1 (1:1000), rabbit anti-cleaved caspase 3 (1:1000); mouse anti-β-actin (1:2000). The blots were then incubated for 1 hr with HRP-conjugated species-specific secondary antibodies (1:5000). Proteins were visualized using ECL substrate (Bio-Rad) and ImageJ was used to quantify densitometry and immunoblot results.

### Migration assay

*In vitro* migration (scratch) assay was carried out in LGACC cells. LGACC cells were seeded in 12-well culture plates in complete media, and incubated overnight. A uniform scratch was made in the center of the well using a micropipette tip, and a baseline image taken of the entire scratch width. Cells were then treated with half of the EC_50_ concentrations determined for cisplatin and AZD4547. After 48 hrs. of treatment, cells were fixed with 0.5% glutaraldehyde and stained with crystal violet for 15 min. Images of the scratch area were taken using Zeiss primovert microscope and the areas of gap (scratch) before and after treatment were analyzed using ImageJ for calculations of the percent bridging by cells.

### Growth curve analysis

Cell numbers and growth rate was measured by means of a (4,5-dimethylthiazol-2-yl)-2,5-diphenyltetrazolium bromide (MTT) assay (Promega, Madison, WI) following the manufacturer's instructions. LGACC cells were treated with either cisplatin, AZD4547 and combination of both cisplatin and AZD4547 for different time intervals in triplicate. MTT reagent was added to the media to cellular NAD(P)H-dependent processing to insoluble formazan for 2 hr. Formazan crystals are then solubilized using 20% SDS solution, and the amount of formazan was measured by absorbance at 570nm using the SpectraMax i3 plate reader. Cell fold over time was then plotted to calculate population doubling times during the linear growth phase of cell expansion.

### Statistics

All observations in this study were analyzed in triplicate and each experiment was repeated three times. Similarly, the results presented are aggregates of several cell lines or patient specimens as indicated. GraphPad Prism (San Diego, CA, USA) was used to generate and analyze data. Dose–response data were analyzed by one-way ANOVA followed by Tukey post hoc comparison of all the means to determine significance. Values are presented as the mean ± SEM of independent experiments. Two-group comparissons were achieved by Student's *t*-test and a p< 0.05 was considered as statistically significant.
